# A proteomic analysis of grain yield-related traits in wheat

**DOI:** 10.1093/aobpla/plaa042

**Published:** 2020-08-24

**Authors:** Sintayehu D Daba, Xiaoqin Liu, Uma Aryal, Mohsen Mohammadi

**Affiliations:** 1 Department of Agronomy, Purdue University, West Lafayette, IN, USA; 2 Purdue Proteomics Facility, Bindley Bioscience Center, Purdue University, West Lafayette, IN, USA

**Keywords:** Differentially abundant proteins, label-free quantitative proteomics, unique and shared proteins, yield-contributing organs

## Abstract

Grain yield, which is mainly contributed by tillering capacity as well as kernel number and weight, is the most important trait to plant breeders and agronomists. Label-free quantitative proteomics was used to analyse yield-contributing organs in wheat. These were leaf sample, tiller initiation, spike initiation, ovary and three successive kernel development stages at 5, 10 and 15 days after anthesis (DAA). We identified 3182 proteins across all samples. The largest number was obtained for spike initiation (1673), while the smallest was kernel sample at 15 DAA (709). Of the 3182 proteins, 296 of them were common to all seven organs. Organ-specific proteins ranged from 148 in ovary to 561 in spike initiation. When relative protein abundances were compared to that of leaf sample, 347 and 519 proteins were identified as differentially abundant in tiller initiation and spike initiation, respectively. When compared with ovary, 81, 35 and 96 proteins were identified as differentially abundant in kernels sampled at 5, 10 and 15 DAA, respectively. Our study indicated that two Argonaute proteins were solely expressed in spike initiation. Of the four expansin proteins detected, three of them were mainly expressed during the first 10 days of kernel development after anthesis. We also detected cell wall invertases and sucrose and starch synthases mainly during the kernel development period. The manipulation of these proteins could lead to increases in tillers, kernels per spike or final grain weight, and is worth exploring in future studies.

## Teaser

We studied the proteome profiles during the kernel development process in wheat. Using proteomics procedure, seven plant organs were quantified for protein abundance. A total of 3182 proteins were identified across all the organs. Two hundred ninety-six proteins were identified in all the organs. This study demonstrated organ-specific detection and abundance of many important wheat proteins that may contribute to tiller formation, spike initiation and kernel development. The findings from this study could pave the way for in-depth study of the different components of yield formation in wheat.

## Introduction

Wheat contributes to nearly one-fifth of the total dietary calories and protein worldwide (Shiferaw *et al.* 2010; Reynolds *et al.* 2012). European Union, China, India and the USA are responsible for 60 % of the global production (Hawkesford *et al.* 2013). Genetic gains, defined as yield increases per unit time, have recently slowed down to <1 % per annum (Reynolds *et al.* 2012), while the consumption of cereals has increased worldwide (Shiferaw *et al.* 2010). For example, in China, wheat consumption increased from 19 million tons in 1962 to 123 million tons in 2012—a 6-fold increase in half a century (Curtis and Halford 2014). Therefore, it is important to identify the barriers and accelerate the genetic gains in wheat and other cereals.

Tiller number is a major agronomic trait in cereal crops affecting plant architecture and grain yield (Xu *et al.* 2017), which influences grain number (GN) per unit area (Sreenivasulu and Schnurbusch 2012). Final grain yield is the product of GN per unit area and kernel weight (KW) (Sreenivasulu and Schnurbusch 2012). The GN, in combination with the size of grains makes the total sink size (or sink strength). The limitation in sink strength is already a well-known bottleneck in modern wheat production (Calderini *et al.* 2006; Reynolds *et al.* 2012; Serrago *et al.* 2013). Yield improvements in the past have generally resulted from increases in GN rather than increases in KW. Understanding the physiology and genetics of sink strength is essential to design high-yielding crosses that could successfully enhance GN, mainly by increasing the number of grains per spike dry matter (Ferrante *et al.* 2012), while maintaining, if not increasing, the contribution of KW in improving yield (Sadras and Lawson 2011; Pask and Reynolds 2013; Aisawi *et al.* 2015).

Evidences provided by Miralles and Slafer (1995) and Acreche and Slafer (2006) suggest that the undesirable correlation between major yield components may not be due to a competition between growing grains. Experimental data showed simultaneous increases of GN and KW in the progeny of a cross between two CIMMYT lines with exceptional yield potential (Bustos-Korts *et al.* 2013; García *et al.* 2013). This cross produced grain yield of up to 16 t ha^−1^ in 3–4 % of the breeding progeny in exceptionally favourable environments (Bustos-Korts *et al.* 2013). This means that the undesirable correlation between the two major yield components may not represent any competition between growing grains after anthesis (Miralles and Slafer 1995; Acreche and Slafer 2006). Therefore, identification of genes that control GN and KW may provide opportunities to improve grain yield.

Grain number is the result of tiller number and number of grains per spike. Several genes were shown to be linked to tiller number and GN or grain yield. This includes low tillering (*tin*) and *TEOSINTE BRANCHED1* (*TB1*) orthologue in wheat (Sreenivasulu and Schnurbusch 2012; Xu *et al.* 2017; Dixon *et al.* 2018); *uniculm* (*vrs1*) and *INT-C* in barley (Li *et al.* 2003; Komatsuda *et al.* 2007; Ramsay *et al.* 2011; Hussien *et al.* 2014); MONOCULM (*MOC1*) in rice (Li *et al.* 2003; Hussien *et al.* 2014); *TEOSINTE BRANCHED 1* in maize (Doebley *et al.* 1997); and multiseed (MSD) mutant in sorghum (Jiao *et al.* 2018).

The development of reproductive growth involves spikelet formation and spike elongation. Identification of genes that derive inflorescence architecture can help improve our understanding and potentially help identify natural variants and their uses in wheat breeding. For example, Schilling *et al.* (2018) extensively discussed the importance of MADS-box genes for reproductive development. In general, the MADS-box genes are key regulators of flowering time, inflorescence architecture, floral organ identity and seed development.

Omics methods are valuable tools to identify genes and proteins that are differentially expressed/abundant in different organs, during different growth stages, and under different environmental conditions. In wheat, proteomics approaches are used in a variety of research such as grain development processes, spatiotemporal expression mapping and disease responses (Zhang *et al.* 2015; Yang *et al.* 2016a, b, 2017; Duncan *et al.* 2017). A recent large-scale proteome map of wheat, reporting on 24 organs and developmental samples was published by Duncan *et al.* (2017), representing a wide range of spatial and temporal organs, without regard to source and sink specificity. Their work aimed at dissecting total proteome and correlation of transcript and protein data for different wheat tissues. Given the large and hexaploid nature of the wheat genome (Appels *et al.* 2018), the task of unbiased and accurate identification of proteins based on masses is challenging. Majority of earlier wheat proteomics studies, Duncan *et al.* (2017) for example, did not use the recent reference genome assembly IWGSC v1.0 (Appels *et al.* 2018). In addition to the IWGSC v1.0 assembly, advances in mass spectrometry (MS)-based proteomics technique now provide an unprecedented opportunity for accurate identification and quantitation of proteins and assigning them to homologous groups in the wheat genome. We sought to identify proteins that are differentially abundant in different organs that contribute to the development of organs that produce tillers, spikes and kernels. These traits enhance sink strength and are physiologically relevant to the determination of grain yield. We conducted this study by employing liquid chromatography-tandem mass spectrometry (LC-MS/MS) analysis.

## Materials and Methods

### Plant growth condition

We obtained seed for accession ‘PI 495816 Cranbrook’ from the National Small Grain Collection, Aberdeen, ID, USA. One hundred seeds of variety ‘Cranbrook’ were germinated on a tray filled with Metro-mix soil (Sun Gro Horticulture Canada Ltd) in the greenhouse condition, with 25 °C day/22 °C night ± 2 °C temperature and 16 h/8 h day/night photoperiod. Seven days after sowing, each seedling was transplanted into a square pot (10 cm × 10 cm × 15 cm). For nutrients, we used a general-purpose greenhouse 24-8-16 N-P-K Miracle-Gro (ScottsMiracle-Gro), by dissolving 5.3 g of Miracle-Gro per litre of water. The nutrient solution was applied to the plants, starting from three-leaf stage, at a rate of 100 cm^3^ per pot, twice a week.

### Experimental design and sample collection

**Figure 1.**
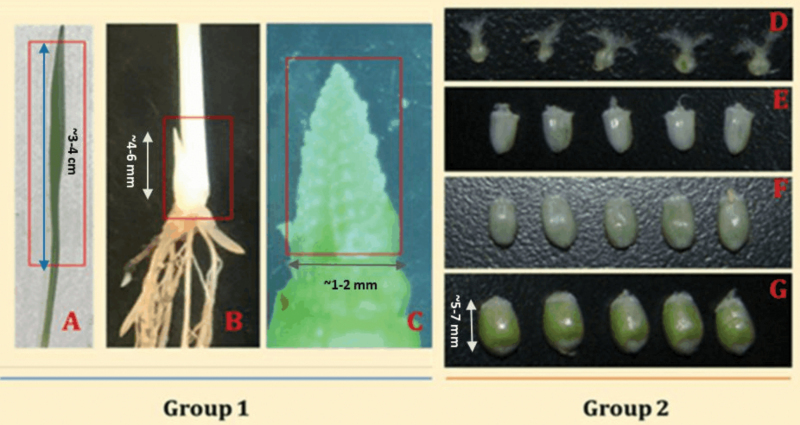
Tissues sampled for the proteomics study, namely; (A) leaf, (B) tillering initiation site, (C) terminal spikelet stage, (D) Ovary, (E) 5 days post anthesis (5DAA), (F) 10 days post anthesis (10DAA), (G) 15 days post anthesis (15DAA). The seven target samples were categorized into **Group 1**, representing leaf, tiller initiation site, and the terminal spikelet sample while **Group 2** represents ovary and progressive kernel development stages. For the leaf sample, the first leaf was sampled when the second leaf just emerging.

This study was designed to identify proteins associated with the following traits: the total number of grains by means of tillering and grains per spike, and the growth of the developing kernels. Therefore, the experimental design consisted of collecting three biological replicates of organs that are involved in these traits. Each biological replicate consisted of organs collected from three independent plants. We collected seven tissue samples. Leaf sample (LS) was taken at the seedling stage, when the first leaf was fully expanded and the second leaf just emerging (Fig. 1A). For LS, we cut a 3-cm segment from the fully developed leaf. Tiller initiation (TI) was taken at tillering stage (Fig. 1B). Tiller initiation for Cranbrook occurred in our greenhouse when plants were in the three-leaf stage. At this stage, we removed the plant gently from the soil and sampled the budding tiller by excising it from the main stem using a sharp cutter (Fig. 1C). The third sample, spike initiation (SI), was taken during the formation of terminal spikelet after a double-ridge stage in the inflorescence structure. Approximately 4–5 weeks after emergence, when plants were at tillering and before stem elongation, the leaves were gently removed from the plant and the very tip of the terminal meristem was excised by using a laboratory blade cutter under binocular. Ovary (OV) samples were taken before anthesis. The kernel development samples were taken successively at 5, 10 and 15 days after anthesis (DAA), named 5, 10 and 15 DAA. The kernel samples at each time point were first collected in polystyrene weighing dish floated in liquid nitrogen. After enough kernels were collected, the samples were wrapped in aluminium foil, and then flash-frozen in liquid nitrogen. For all the other samples, we wrapped the samples immediately in aluminium foil and flash-frozen in liquid nitrogen. All the sampled tissues were kept in deep freezer until processed in the proteomics facility. No other standards or internal references were used in this study.

In this study, we also monitored accumulation of dry matter in the developing kernels. Unlike the proteomics experiment where three time points were studied, we sampled developing kernels at five developmental stages 5, 10, 15, 20 and 25 DAA. At each time point, three spikes were sampled, kernels from each spike were harvested separately, counted and weighed to obtain fresh weights (*W*_f_). Then, we oven-dried the developing kernels for 24 h at 60 °C and measured the dry weight (*W*_d_). The averages of *W*_d_ and *W*_f_ per single kernel were expressed in milligram (mg). The difference between the *W*_f_ and *W*_d_, expressed as percentage, was used as a measure of moisture content.

### Protein extraction and LC-MS sample preparation

Biological samples were homogenized in 400 µL of 20 mM Tris–HCl, pH 7.5, 100 mM NaCl, 1 mM EDTA (ethylene diamine tetraacetic acid), 5 % glycerol and 0.5 mM DTT (dithiothreitol) using Precellys® 24 Bead Mill Homogenizer (Berti) at 4487 g for 30-s cycles. The tissue lysates were centrifuged at 20 817 g for 15 min at 4 °C, and the supernatant was transferred to new tubes. The insoluble pellet fractions were solubilized in 400 µL of 8 M urea and were incubated at room temperature for 1 h with continuous mixing. Then, the solution was centrifuged at 20 817 g for 15 min at 4 °C to remove any undissolved pellets and cell debris. Proteins both in soluble and insoluble fractions were precipitated using five volumes (v/v) of cold (−20 °C) acetone and incubated overnight at −20 °C. Precipitated proteins were pelleted by centrifugation at 20 817 g for 15 min at 4 °C. Protein pellets were washed once with 80 % cold (−20 °C) acetone and redissolved in 8 M urea. The bicinchoninic acid (BCA) assay was used to determine protein concentration in each sample with bovine serum albumin (BSA) as a standard (Kapoor *et al.* 2009).

About 50 µg protein from each sample was reduced by incubating with 10 mM DTT at 37 °C for 45 min, and cysteine alkylated with 20 mM iodoacetamide (IAA) in the dark for 45 min at room temperature. Then, the solution was incubated in 5 mM DTT for 20 min at 37 °C to scavenge residual IAA. Proteins were digested using sequencing-grade trypsin and Lys-C mix from Promega at a 1:25 (w/w) enzyme-to-protein ratio and 37 °C temperature overnight. The digested peptides were cleaned using C18 silica micro-spin columns from The Nest Group Inc. using the manufacturer’s protocol. Peptides were eluted using 80 % acetonitrile containing 0.1 % formic acid (FA). The samples were vacuum-dried and resuspended in 3 % acetonitrile and 0.1 % FA. Peptide concentration was determined by BCA assay using BSA as standard. The concentration of peptides was adjusted to 0.2 µg µL^−1^, soluble and insoluble samples were mixed and 5 µL (1 µg total peptide) was used for LC-MS/MS analysis as described below.

### LC-MS/MS data acquisition

Samples were analysed by reverse-phase LC-ESI-MS/MS system using the Dionex UltiMate 3000 RSLC nano System coupled with the Q Exactive High Field (HF) Hybrid Quadrupole Orbitrap MS (Thermo Fisher Scientific). Peptides were loaded onto a trap column (300 µm internal diameter (ID) × 5 mm) packed with 5 µm 100 Å PepMap C18 medium, and then separated on a reverse-phase column (15 cm long × 75 µm ID) packed with 3 µm 100 Å PepMap C18 silica (Thermo Fisher Scientific). All the MS measurements were performed in the positive ion mode and using 120-min LC gradient. Mobile phase solvent A was 0.1 % FA in water and solvent B was 0.1 % FA in 80 % acetonitrile. Peptides were loaded to the trap column in 100 % buffer A for 5 min at 5 µL min^−1^ flow rate, and eluted with a linear 80-min gradient of 5–30 % of buffer B, then changing to 45 % of B at 91 min, 100 % of B at 93 min at which point the gradient was held for 7 min before reverting to 95 % of A at 100 min. Peptides were separated from the analytical column at a flow rate of 300 nL min^−1^. The column temperature was maintained at 50 °C. The mass spectrometer was operated using standard data-dependent mode. Mass spectrometry data were acquired with a Top20 data-dependent MS/MS scan method. The full-scan MS spectra were collected in the 300–1650 m/z range with a maximum injection time of 30 ms, a resolution of 120 000 at m/z 200, spray voltage of 2 and AGC target of 1 × 10^6^. Three biological samples were analysed for each organ. Fragmentation of precursor ions was performed by high-energy C-trap dissociation (HCD) with the normalized collision energy of 27 eV. The MS/MS scans were acquired at a resolution of 15 000 at m/z 200 with an ion-target value of 1 × 10^5^ and a maximum injection of 60 ms. The dynamic exclusion was set at 30 s to avoid repeated scanning of identical peptides. The instrument was calibrated at the start of each batch run and then in every 72 h using a calibration mix solution (Thermo Scientific). The performance of the instrument was also evaluated routinely using *Escherichia coli* digest.

### Data analysis

All LC-MS/MS data were analysed using MaxQuant software (v. 1.6.0.16) 17-19 with built-in Andromeda search engine. We searched the MS/MS spectra against wheat (*Triticum aestivum*) IWGSC v1.0 genome assembly, which was recently published by Apples *et al.* (2018), and is publicly available at (https://urgi.versailles.inra.fr/download/iwgsc/IWGSC_RefSeq_Annotations/v1.1/).

The IWGSC v1.0 assembly contains 107 891 high-confidence genes. The database search was completed by considering the minimum amino acid length of six, a precursor mass tolerance of 10 ppm and a MS/MS fragment ion tolerance of 20 ppm. Database search was performed with enzyme specificity for trypsin and Lys-C, allowing up to two missed cleavages. Oxidation of methionine (M) was defined as a variable modification, and carbamidomethylation of cysteine (C) was defined as a fixed modification for database searches. The ‘unique plus razor peptides’ were used for peptide quantitation. The false discovery rate (FDR) of both peptide spectral match (PSM) and proteins identification was set at 0.01. Proteins labelled either as contaminants or reverse hits were removed from the analysis. Similarly, proteins identified without any quantifiable peak (0 intensity) and those identified by a single MS/MS count were also removed from downstream analyses. We only kept proteins that were detected in at least two biological replicates. We also consider proteins with MS/MS counts > 4 for further downstream analyses.

For downstream analysis, we used the InfernoRDN (formerly known as DanTE) bioinformatics software (Polpitiya *et al.* 2008) to produce a correlation heat map and 3D plot of the first three principal components (PCs). To highlight the number of unique or general proteins across different samples, we drew Venn diagrams using the InteractiVenn (Heberle *et al.* 2015) online tool. For quantitative abundance analysis, we used two-layer criteria. First, we compared mean abundance values, in log2 scale, by using *t*-test and screened proteins with adjusted *P*-value < 0.01. Then, further screened proteins that differed in abundance level by log2 fold change > 3. These criteria allowed identifying differentially abundant proteins (DAPs). To functionally group proteins, we used the Cluster of Orthologous Groups of protein database (COGs, http://www.ncbi.nlm.nih.gov/COG). We also used AgriGO (bioinfo.cau.edu.cn/agriGo/) and wheat Ensembl BLAST (plants.ensembl.org/Triticum_aestivum/Info/index) to make further functional characterization.

## Results

### Overview of the experiment

**Figure 2.**
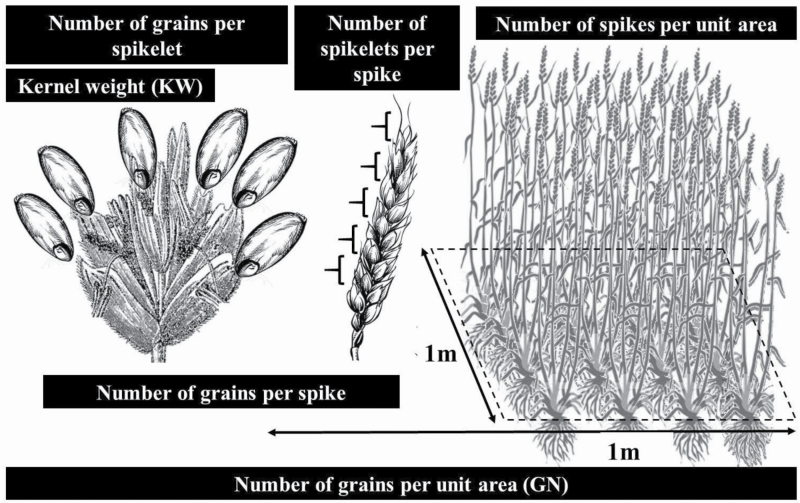
Hierarchical structure of yield component traits showing number of spikes per unit area (right), number of spikelets per spike (middle) and the number of grains per spikelet (left), altogether representing number of grains per unit area (GN). The weight of each grain known as kernel weight (KW).

**Figure 3.**
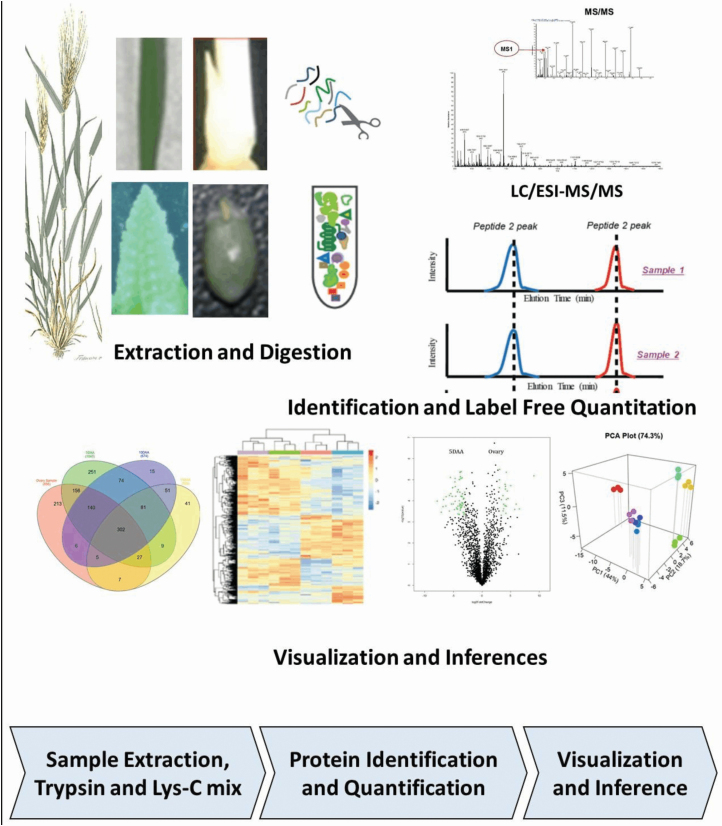
Proteomic workflow: tissues were flash-frozen in liquid nitrogen. Proteins were extracted and digested into fragments using a combined action of trypsin and Lys-C mix. The protein fragments were separated using liquid chromatography (LC) and then quantified by mass spectrometry (MS). The MS/MS spectra were searched against the IWGSC v1.0 genome assembly of wheat (*Triticum aestivum*) recently published by Apples *et al.* (2018). Finally, label-free quantitation values were used in log2 scale for *t*-test comparisons.

Within the framework of yield formation (Fig. 2), it is useful to uncover sets of genes and proteins that are functional for the development of these organs. For example, it is important to know which genes and proteins are involved in the initiation and development of tillers in wheat seedlings. To identify these genes, we analysed wheat organs by using a quantitative proteomics approach. Seven plant organs were sampled (Fig. 1) including LS, TI, SI, OV, 5 DAA, 10 DAA and 15 DAA. We hypothesized that TI represents enrichment for proteins that are abundant (and therefore the closest approximation of) tiller formation. The SI sample provides enrichment for proteins involved in SI and determination of the number of kernels per spike. The OV and the subsequent three kernel development samples (5, 10 and 15 DAA) provide enrichment of proteins that are abundant during kernel formation and progression towards developmental stages, and, therefore, critical for determination of kernel size and weight. Based on this rationale, we studied these yield forming organs via label-free LC-MS/MS-based proteome quantification according to schematic proteomic workflow presented in Fig. 3 to identify and quantify the intensity of proteins for each sample.

### Protein identification across different organs

A total of 15 244 peptides were identified across all samples by LC-MS/MS analysis **[see**  **Supporting Information—Table S1****]**. After filtering, 3182 proteins were retained and used for downstream analyses **[see**  **Supporting Information—Table S2****]**. The raw MS/MS data were uploaded to MASSIVE Data Repository (https://massive.ucsd.edu/), and are accessible under accession MSV000083833. The largest number of expressed proteins were encoded by genes located on subgenome D (*n* = 1550), as per IWGSC v1.0, followed by *n* = 793 proteins were encoded by genes located on subgenome B, and, finally, *n* = 776 proteins by the genes located on subgenome A. For example, 272 of the proteins were encoded by genes located on chromosome 2D while 95 proteins were encoded by genes located on chromosome 6A. We also identified 63 proteins that matched to the genes on IWGSC v1.0 but not yet associated to any of the chromosomes, and so, are unmapped.

**Figure 4.**
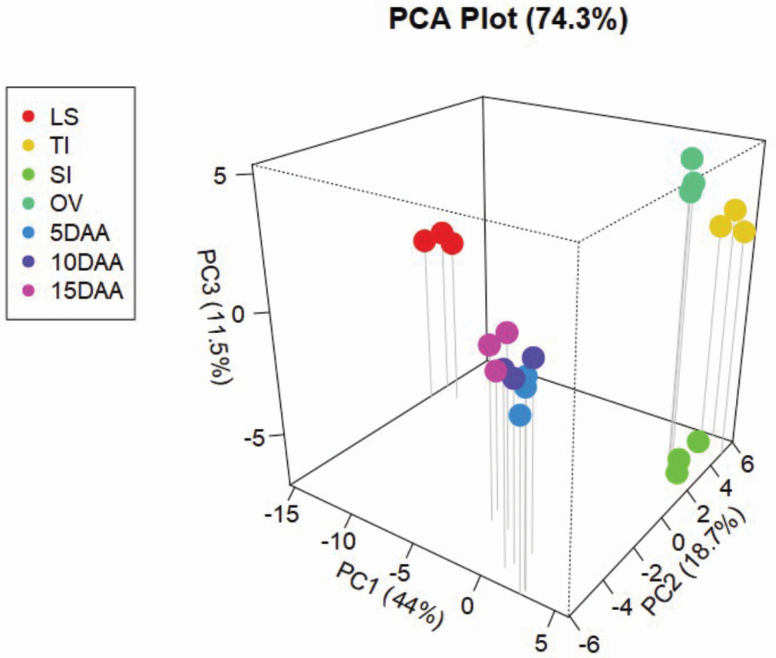
The 3D plot of the first three principal components of the protein intensities of 7 tissue samples. LS = Leaf sample; TI = tiller initiation; SI = spike initiation; OV = ovary; 5DAA = 5 days after anthesis; 10DAA = 10 days after anthesis; and 15DAA = 15 days after anthesis.

Principal component analysis (PCA) was performed to summarize label-free quantitation (LFQ) measurements into a 3D space (Fig. 4). We applied PCA to all proteins that were quantified in each organ sample. The first PC, which accounted for 44 % of variation, created three groups. The first group, on the far left, had only one organ and which was LS. The second group on the far right included OV, SI and TI. The middle group included the three stages of kernel development, i.e. 5, 10 and 15 DAA. The second PC, accounting for 18.7 % of variation, grouped the organs almost in the same patterns. However, the third PC, which accounted for 11.5 % of variation, mainly separated the SI (on the bottom across the PC3 axis, Fig. 4) from OV and TI (on the top, across the PC3 axis). Altogether, all three components accounted for 74.3 % of the total variation. In addition, the close clustering of the three biological replicates of each organ together, revealed by PC analysis (Fig. 4), indicated the LC-MS/MS analysis and peptide quantification were largely reproducible, which is a prerequisite for accurate label-free protein quantitation.

### Unique and shared proteins across different organs

**Figure 5.**
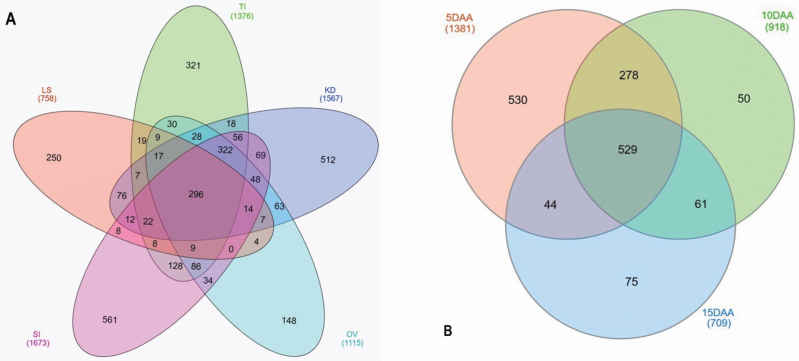
Venn diagrams showing the distribution of common and uniquely abundant proteins across samples. (A) Protein distribution in five groups with kernel development stages combined and (B) proteins only across the three kernel development stages. LB = leaf sample; SI = spike initiation; TI = tiller initiation; OV = ovary; 5 DAA = 5 days after anthesis; 10 DAA = 10 days after anthesis; and 15 DAA = 15 days after anthesis.

Two Venn diagrams (Fig. 5A and B) represent the shared proteins in all organs and the unique proteins identified in each organ. The list of genes and their corresponding proteins in each of the sampled tissues were given in **Supporting Information—Table S3**). The lowest number of proteins was identified in 15 DAA sample (*n* = 709) and the highest number of proteins was identified in SI sample (*n* = 1673). For simplicity, data for all three stages of kernel development (5, 10 and 15 DAA) were pooled together and reflected in the first Venn diagram, which represents five groups (Fig. 5A). In total, 296 shared proteins were identified across all these samples. The sample with the greatest number of unique proteins was SI (*n* = 561). In contrast, OV showed the lowest number (*n* = 148) of unique proteins. A total of 529 proteins were shared by 5, 10 and 15 DAA (Fig. 5B). The 5 DAA sample had the greatest number (*n* = 530) of unique proteins and the 10 DAA had the lowest number (*n* = 50) of unique proteins.

In TI, a total of 231 proteins were uniquely identified, which were enriched for glutathione-S-transferase, lipid transfer proteins, heat shock proteins, defensins, ferredoxin, actin-related proteins, peroxidases, histones, proteasomes and ribosomal proteins. The unique proteins of the SI sample (*n* = 561) were enriched for RNA processing, ubiquitin-like proteins, heat shock proteins and other transcription factors, as well as several cell division-associated proteins such as mitotic checkpoint protein, mitotic spindle-organizing protein and sister chromatid cohesion protein. Similarly, unique proteins in 5 DAA were enriched for carbohydrate-degrading enzymes and cell division-related proteins such as starch synthase and branching, expansins, defensins, protease inhibitors, subtilisin-like proteins, peroxidases, gibberellin-regulated protein, actin-like proteins and DELLA protein. The 10 DAA unique proteins included acid phosphatase, invertase, storage proteins and a *TERMINAL FLOWER 1-like* protein. The 15 DAA unique proteins were considerably enriched with storage proteins and, to a lesser extent, with xylanase inhibitors, lipid transfer proteins, thaumatin-like proteins, vicilin-like proteins and defensins. Interestingly, one sucrose synthase protein, grain softness proteins, dimeric α-amylase inhibitors and desiccation-related proteins were also exclusively expressed in kernels sampled at 15 DAA.

### Quantitative proteome expression and organ comparisons

#### Expression profiles in TI and SI (as compared with LS).

We compared TI with LS by using a *t*-test to identify DAPs. There were 200 DAPs with increased abundance and 147 DAPs with decreased abundance in the TI compared to the LS (Table 1; **see**  **Supporting Information—Table S4**). Proteins related to translation, ribosomal structure and biogenesis; amino acid transport and metabolism; post-translational modification; and chromatin structure and dynamics were higher and those related to chaperone and heat shock proteins, electron transport, lipid biosynthesis, energy production and conversion; and carbohydrate transport and metabolism were relatively lower in TI compared to LS.

**Table 1.** The number of differentially abundant proteins (DAPs) (significantly high and low proteins) in tiller initiation (TI), spike initiation (SI), and kernel development stages. The TI and SI were compared against leaf sample while the kernel development stages were compared with ovary sample.

	TI vs. LS	SI vs. LS	OV vs. 5 DAA	OV vs. 10 DAA	OV vs. 15 DAA
Increased abundance	200	313	57	17	67
Decreased abundance	147	206	24	18	29

Similarly, we compared SI with LS by using *t*-test to identify DAPs, where we identified 313 DAPs with enhanced abundance and 206 DAPs with decreased abundance in the SI compared to the LS (Table 1; **see**  **Supporting Information—Table S4**). The majority of the proteins with enhanced abundance in SI were grouped as translation, post-translational modification, amino acid transport and metabolism, chromatin structure and dynamics, carbohydrate transport and metabolism, and energy production and conversion.

#### Considerable grain size increase occurred until 10 DAA.

**Figure 6.**
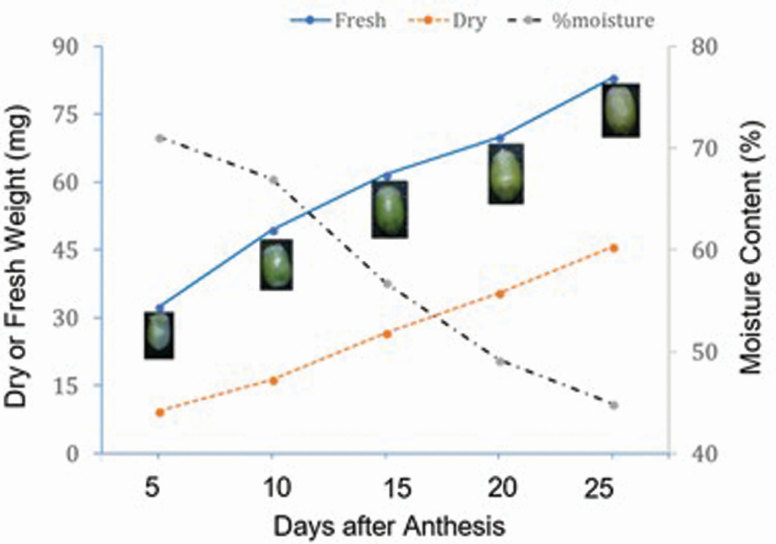
Change in kernel weight and moisture content during kernel development in cultivar ‘Cranbrook’.

To understand the pattern of kernel development, we measured fresh and dry weights of the grains taken at five developmental stages (5, 10, 15, 20 and 25 DAA). Both fresh and dry weights increased consistently (Fig. 6). The dry weight increased by 3.5-fold between 5 and 25 DAA. The moisture content dropped sharply, particularly beyond 10 DAA. Interestingly, the size of the grain also remained constant beyond 10 DAA. These results could imply that, in the variety tested (Cranbrook), the first 10 DAA was the period of a significant increase in grain size.

#### Expression profiles in 5, 10 and 15 DAA (as compared with OV).

For the comparison of protein expression levels during kernel development stages, we compared each stage with OV sample, with the same two-step criteria for identifying DAPs. At 5 DAA, a total of 81 DAPs were identified **[see**  **Supporting Information—Table S4****]**, of which 57 DAPs showed enhanced abundance levels and 24 DAPs showed decreased abundance levels than the OV (Table 1). About 53 % (30 proteins) of DAPs with enhanced abundance in 5 DAA were grouped as amino acid transport and metabolism, carbohydrate transport and metabolism, and energy production and conversion. Approximately 41 % of the DAPs with decreased abundance in 5 DAA were involved in translation and transcription. At 10 DAA, a total of 35 DAPs were identified (Table 1; **see**  **Supporting Information—Table S4**), of which 17 DAPs showed enhanced abundance than OV while 18 DAPs showed decreased abundance than OV. About 67 % of DAPs with enhanced abundance in 10 DAA were involved in carbohydrate transport and metabolism, post-translational modification, protein turnover, chaperones, and amino acid transport and metabolism. Approximately 60 % of the DAPs with decreased abundance in 10 DAA were involved in transcription, carbohydrate transport and metabolism, translation, inorganic ion transport and metabolism, and defence mechanisms. In total, 96 DAPs were identified in kernels sampled at 15 DAA compared to OV (Table 1; **see**  **Supporting Information—Table S4**), of which 67 DAPs showed enhanced abundance levels while 29 DAPs exhibited decreased abundance levels. Approximately 76 % of DAPs with enhanced abundance than OV in 15 DAA were functionally grouped as storage proteins such as glutenin, globulins and gliadins; and carbohydrate transport and metabolism such as β-amylase and α-amylase inhibitors. The DAPs with decreased abundance in 15 DAA compared to OV were involved in translation, ribosomal structure and biogenesis; energy production and conversion; chromatin structure and dynamics; and transcription. Proteins unique to 5, 10 and 15 DAA samples were involved in carbohydrate metabolic process, nutrient reservoir activity, cellulose activity, cell wall organization and fatty acid biosynthesis.

## Discussion

Grain yield depends in part on proteins that function in organ biogenesis, development of tillers, spikes and kernels, carbohydrate metabolism and nutrient storage. Expression of genes can be evaluated via transcriptome studies. Proteome analysis can be studied via 2D gel electrophoresis as well as shotgun proteomics approaches. Previous proteomic studies were carried out before the publication of wheat genome assembly (Jiao *et al.* 2018), and mostly relied on published genomes of closely related species. Therefore, none those studies could be matched with the exact homoelogous genes among the three wheat subgenomes.

Duncan *et al.* (2017) presented the most comprehensive wheat proteomics study to this date. They completed a proteomics search on 24 organs. However, their effort was without regard to any specific phenotypic goal. For example, they produced a large specio-developmental proteomic map, which showed what proteins were expressed in which organ. In contrast, our study targeted organs that are specifically related to GN and KW. Our organ sampling strategy is different from theirs because of inclusion of tillering site and inflorescence initiation site in our study and a continuum spectrum of kernel development from ovary to later stages of kernel development (e.g. 15 DAA). Secondly, for MS/MS search, Duncan *et al.* (2017) used a protein database containing the protein sequences from the IWGSC1.0 + popseq PGSB/MIPS (Chapman *et al.* 2015) version 2.2 annotation. We used the IWGSC v1.0 wheat genome assembly published in 2018 (Appels *et al.* 2018), which is the complete assembly. Lastly, Duncan *et al.* (2017) used label-free spectral counts for protein quantification but spectral count-based proteomics is semi-quantitative. It has long been established that protein quantification using MS1 intensity greatly enhances quantitative accuracy and dynamic range compared to spectral counts (Bondarenko *et al.* 2002). For this reason, in this study, we used label-free MS1 intensity-based quantitative proteomics. We discuss these identified proteins under three categories of organ biogenesis, carbohydrate metabolism and storage proteins. Similar to other studies that are conducted in controlled environment, this data may not be predictive of proteomic responses under natural field conditions. The non-homogenous nature of field, and interaction of the microclimate by unsynchronized growth stages of plants can all be potential cause for the lack of transferability results to field conditions. However, our study provided insight into specific proteins and their corresponding encoding genes (Table 2). These genes, after a thorough study, could be incorporated in elite breeding lines that will be tested for their effect in the actual field condition.

**Table 2.** Gene ID, annotation, number of peptides per protein, protein score, and Label Free Quantitation (LFQ) averages across the three biological replicates of each tissue type. Tissues are leaf blade (LB), tiller initiation (TI), spike initiation (SI), ovary (OV) before anthesis, and three progressive kernel development stages of five, ten, and fifteen days after anthesis, named as 5DAA, 10DAA, and 15DAA. FDR for protein identification was set to 1%.

Gene ID	Annotation	Unique + razor peptide counts	Protein score	LB	TI	SI	OV	5 DAA	10 DAA	15 DAA
TraesCS4A01G485400	Acid invertase 1	1	78.096	0	0	0	6507667	0	0	0
TraesCS2B01G311900	Cell wall invertase	1	53.754	0	0	0	0	14909000	0	0
TraesCS2A01G295400	Cell wall invertase	12	7969	0	0	0	0	697653333	285276667	0
TraesCS4D01G323800	Expansin-like protein	1	7589	0	0	0	0	15423667	0	0
TraesCS3D01G338600	Expansin	2	1501	0	0	0	101763333	0	0	0
TraesCS1D01G215400	Expansin protein	5	8271	0	0	0	0	141236667	48413333	0
TraesCS4B01G327100	Expansin-like protein	4	8723	0	0	0	0	212326667	341146667	107060000
TraesCS2D01G361800	Proliferating cell nuclear antigen	13	2609	421443333	4605166667	7952966667	2081900000	688806667	513666667	353870000
TraesCS4D01G267800	Cell division cycle protein 48-like protein	30	9358	0	199080667	1234366667	325096667	1356760000	397300000	84037000
TraesCS5D01G314000	Cell division cycle 5-like protein	7	6775	0	18002000	136700000	0	0	0	0
TraesCS2D01G488700	Cell division protein ftsZ	7	7762	0	89712000	158176667	0	42246333	0	0
TraesCS7B01G269000	Cell division cycle and apoptosis regulator protein 1	2	1617	0	0	40253333	0	0	0	0
TraesCS2D01G292200	Cell division protein ftsZ	3	132.01	0	0	0	0	78183000	137990000	32470667
TraesCS1D01G453600	Argonaute	3	5092	0	0	14506000	0	0	0	0
TraesCSU01G065300	Argonaute	2	7107	0	0	5056000	0	0	0	0
TraesCS2B01G194200	Sucrose synthase	3	1739	0	0	0	0	368466667	906160000	2053586667
TraesCS2A01G168200	Sucrose synthase	33	7356	0	9459000	0	283148667	2696286667	5025100000	8804000000
TraesCS7A01G189000	Starch synthase, chloroplastic/amyloplastic	22	6148	0	0	0	0	1012010000	1363776667	517848000
TraesCS7D01G064300	Starch synthase, chloroplastic/amyloplastic	23	9849	0	0	0	0	3469100000	5835333333	4703233333
TraesCS5A01G464500	Alpha-amylase	12	7578	0	0	0	0	1219750000	845143333	753446667
TraesCS7A01G383900	Alpha-amylase	15	6552	0	0	0	328546667	2520300000	1055736667	1090773333
TraesCS2A01G215100	Beta-amylase	2	2004	0	0	0	0	71195000	0	0
TraesCS4B01G393500	Beta-amylase	8	7526	0	0	0	0	24626000	516360000	5438866667
TraesCSU01G032700	Beta-amylase	19	9394	0	0	0	0	105910000	4329770000	21142366667
TraesCS7D01G083700	Dimeric α-amylase inhibitor	3	217.02	0	0	0	0	311396667	4266500000	8435500000
TraesCS3B01G111200	Dimeric α-amylase inhibitor	6	3125	0	0	0	0	1055233333	11218333333	40279333333
TraesCS7B01G072000	Alpha-amylase inhibitor protein	1	3223	0	0	0	0	220766667	653893333	6300433333
TraesCS7D01G168000	Alpha-amylase inhibitor protein	4	6383	0	0	0	0	66746667	550763333	4586966667
TraesCS5D01G004300	Puroindoline-b	3	6998	0	0	0	0	0	122886667	680096667
TraesCS5D01G004000	Grain softness protein	3	8245	0	0	0	0	0	2837043333	7778266667
TraesCS5A01G003300	Grain softness protein	4	9358	0	0	0	0	105343667	3060623333	9518133333
TraesCS5B01G003500	Grain softness protein	1	117.04	0	0	0	0	0	0	29034000
TraesCS1A01G317500	High-molecular-weight glutenin subunit	2	171.92	0	0	0	0	0	64856667	1380400000
TraesCS1B01G330000	High-molecular-weight glutenin subunit	2	156.81	0	0	0	0	0	61260000	1118073333
TraesCS1D01G317300	High-molecular-weight glutenin subunit	6	8697	0	0	0	0	0	886550000	3685733333
TraesCS1A01G008000	Low-molecular-weight glutenin subunit	2	2848	0	0	0	0	236596667	1644000000	10351166667
TraesCS1B01G013500	Low-molecular-weight glutenin subunit	5	2087	0	0	0	0	99570333	4375070000	21306666667
TraesCS1D01G000200	Low-molecular-weight glutenin subunit	4	2612	0	0	0	0	143736667	3398666667	22579000000
TraesCS6A01G049800	Alpha-gliadin	1	78	0	0	0	0	0	0	673000000
TraesCS1A01G007700	Gamma-gliadin	3	7406	0	0	0	0	33373333	1993100000	14268666667
TraesCS7D01G031700	Gliadin-like avenin	5	3365	0	0	0	0	71090000	562143333	3430333333
TraesCS1A01G066100	11S globulin seed storage protein	2	3002	0	0	0	0	0	62060000	0
TraesCS1B01G059000	11S globulin seed storage protein 2	3	7785	0	0	0	110496667	0	194270000	125760000
TraesCS1D01G067100	11S globulin seed storage protein	16	9858	0	0	0	0	57656667	2631383333	1741700000
TraesCS5A01G432100	Globulin-1	7	8313	0	0	0	0	0	0	432464000
TraesCS5B01G434100	Globulin-1	3	6876	0	0	0	0	0	0	310840000
TraesCS5D01G440000	Globulin-1	12	6635	0	0	0	0	0	36393333	999953333

### Organ biogenesis

A proliferating cell nuclear antigen (PCNA), TraesCS2D01G361800, on chromosome 2D, was identified with nearly 11-fold and 19-fold higher abundance in TI and SI tissues than in LS, respectively. Proliferating cell nuclear antigen and *TEOSINTE BRANCHED1* (*TB1*) are members of TCP transcription factor family (Li 2015). While increased expression of *TB1* restricts the outgrowth of axillary buds into lateral branches in maize and wheat (Doebley *et al.* 1997; Lewis *et al.* 2008), members of PCNA were shown to promote cell proliferation and organ growth in rice (*Oryza sativa*) (Li 2015). Unlike *TB1*, PCNA acts as a positive regulator of TI. Therefore, we predict that overexpression of this gene could result in increases in final tiller number in wheat. We identified two Argonaute proteins, TraesCS1D01G453600 and TraesCSU01G065300, that were solely expressed in SI sample, and not detected in other samples. Argonaute proteins were shown to play a variety of roles in plant development. As loss of function of the B subgenome *ARGONAUTE1d* homoeologous was shown to produce shorter spikes and fewer grains per spike than wild-type controls (Feng *et al.* 2017). Other roles suggested for Argonaute proteins are seed development and grain size and width. For example, *AGO802* was shown (Singh *et al.* 2013) to play an important role in seed development, and preferentially expressed in wheat embryos. The *OsAGO17* was shown to positively regulate grain size and weight in rice (Jun *et al.* 2019).

We also identified four expansin-related genes, three of which, i.e. TraesCS4D01G323800, TraesCS1D01G215400 and TraesCS4B01G3271000, were mainly expressed during kernel development, particularly in the first 10 DAA. Expansins are important proteins for cell wall loosening and cell enlargement (Cosgrove 2000; Lin *et al.* 2005; Marowa *et al.* 2016). Lizana *et al.* (2010) observed that *TaExpA6* accumulates in the early grain development period, implying their role in determination of grain size in wheat. Castillo *et al.* (2018) have shown that expansins are involved in the extension of grain tissue, and their expression is associated with the grain size of sunflower.

Our study identified several proteins present during kernel development and ovary. For example, a cell wall invertase encoded by TraesCS2A01G295400 was found to be highly expressed at early kernel development stage. Invertases, that cleave sucrose into two monosaccharides, mainly appear in two isoforms of vacuolar (acid invertases) and extracellular (cell wall invertases) in plants. Invertases were shown to have a role in the control of cell differentiation and plant development (Silva and Ricardo 1992; Sturm 1999). For example, in a study on water-soluble carbohydrate and remobilization to the grains, significant difference of acid invertase activity was reported between wild and cultivated wheats (Yadhu *et al.* 2015). Functional analyses have shown that the rice *GRAIN INCOMPLETE FILLING 1* gene encodes a cell wall invertase which has a role in carbon partitioning during early grain filling (Wang *et al.* 2008). Wang and Ruan (2012) also proposed the involvement of cell wall invertase in early grain-filling period in cotton. Several other studies have reported the importance of cell wall invertase in kernel development in different cereal crops such as maize (Chourey *et al.* 2006), wheat (Ma *et al.* 2012), barley (Weschke *et al.* 2003) and rice (Hirose *et al.* 2002; Wang *et al.* 2008). While our finding is not a new discovery, the exact identification of the subgenome-specific copy of the invertases identified in this study, along with the availability of complete genomic sequences, will allow future functional analyses in wheat.

### Carbohydrate metabolism

The last step of kernel growth, after cell division and extension, is starch deposition, and the amount of starch synthesis and deposition is critical to kernel development and size (Edurne *et al.* 2003). Sucrose is the main transportable carbohydrate into non-photosynthetic organs. The products of sucrose breakdown are crucial for starch biosynthesis in the amyloplast of the sink organ (Edurne *et al.* 2003). Sucrose that is destined for sink organs is either cleaved by sucrose synthase to yield UDP-glucose and fructose, or by invertase to yield glucose and fructose (Spielbauer *et al.* 2006). Sucrose synthase is a key enzyme in plant sucrose catabolism that enables sucrose mobilization into multiple pathways involved in metabolic, structural and storage functions (Subbaiah *et al.* 2007). Previously, Subbaiah *et al.* (2007) isolated three orthologues of *Sucrose Synthase 2* (*TaSus2*) and mapped them to the wheat chromosomes 2A, 2B and 2D. We identified concerted co-expression of sucrose synthase and starch synthase during kernel development. The sucrose synthases encoded by two wheat genes on chromosomes 2A and 2B (Jiang *et al.* 2011) were at higher abundance during the three kernel development periods than during ovary development, with the highest abundance observed at 15 DAA.

Grain endosperm is composed of starch that is synthesized and accumulated during grain-filling period (Dupont and Altenbach 2003; Ma *et al.* 2014; Huang *et al.* 2016). A strong relationship was observed between starch biosynthesis and grain yield in wheat and maize (Irshad *et al.* 2019). The activity of starch synthase was shown to be the highest at 10–20 DAA (Fahy *et al.* 2018), and that starch accumulation rates are correlated with the activities of key enzymes (Emes *et al.* 2003; Jiang *et al.* 2003). Our study identified the expression of several starch synthase enzymes during kernel development. The expression of sucrose and starch synthesis genes were studied in several other crops such as rice, maize and barley (Spielbauer *et al.* 2006; Hirose *et al.* 2008; Liu *et al.* 2008; Barrero-Sicilia *et al.* 2011). For future follow-up studies, we suggest collecting more biological tissues that allow analysis of starch and other soluble carbohydrates in order to correlate protein expression with starch accumulation.

The starch synthase enzymes synthesize starch that is needed for grain development and growth, and, finally, yield (Parry *et al.* 2011). While starch synthase synthesizes starch by mobilizing products of sucrose metabolism to sink organs, amylases reverse these reactions to degrade starch and provide energy to growing embryos during and after germination. Interestingly, concurrent with expression of starch synthases, we identified several members of the α-amylase and β-amylase protein families.

There are three possible explanations for this co-expression of starch synthases and amylases. The first possible explanation is that the activity of amylases may require post-translational modification such as phosphorylation and thus protein abundance alone is not sufficient for degradation activity. Perhaps phosphorylation only takes place during germination and not during kernel development. One experimental evidence supporting this explanation is that, Dong *et al.* (2015), using 2D differential gel electrophoresis, found that β-amylase was phosphorylated in germinating wheat seeds. The second explanation is that starch synthases and amylases may be expressed in tissues that are spatially different and hence do not act antagonistically. Barrero *et al.* (2013) reported that α-amylase is synthesized by the scutellum and adjacent aleurone, while starch synthase is expressed in endosperm cells. The third explanation is that it has been shown that α-amylase activity increased again during germination to levels much higher than those detected during kernel development (Thévenot *et al.* 1992). Therefore, it is possible that starch degradation requires enzyme activities much higher than the levels observed during kernel development. Therefore, accumulation of amylases during kernel development, by itself, may not be sufficient to drive starch degradation.

### Storage proteins

Mature wheat grains contain 8–20 % protein (Dupont and Altenbach 2003). One group of these proteins, i.e. puroindolines, is responsible for kernel hardness (Giroux and Morris 1997, 1998). Hard grain phenotype results in courser flour with greater abundance of damaged starch granules and their interaction with lipid-binding proteins on the surface of starch granules (Glenn *et al.* 1991; Giroux and Morris 1997; Bhave and Morris 2008), resulting in absorption of more water compared to grains with soft endosperm (Matus-Cadiz *et al.* 2008). One aspect of kernel growth and development is the grain texture. For example, the degree of softness or hardness of the endosperm in wheat is controlled by puroindoline-a (*Pina-D1*), and puroindoline-b (*Pinb-D1*), which are both located at the *Ha* locus (Lillemo *et al.* 2006; Bhave and Morris 2008) on the distal end of the short arm of chromosome 5D. In addition to puroindoline genes, *Grain Softness Protein* (*GSP*) is located on *Ha* locus. However, to date, strong evidence is lacking for its contribution to kernel hardness phenotype. Our study revealed expression of puroindoline and grain softness proteins during kernel development.

In addition to texture-related proteins, albumins, globulins, gliadins and glutenins are major protein components of kernels (Dwivedi *et al.* 2013). Majority of proteins identified during 15 DAA are related to storage proteins such as glutenins, gliadins and globulins. For example, this study identified increasing trends of expression of 26 gliadins, 12 globulins, 8 avenins, and 12 high- and low-molecular-weight glutenins towards later stages (10 and 15 DAA) of kernel development. Albumins and globulins of wheat endosperm represent ~25 % of total grain proteins (Merlino *et al.* 2009). The dough-making quality of wheat grains is associated with glutenin and gliadin composition and properties (Huebbner and Wall 1976; Bottomley *et al.* 1982). During dough development, glutenin and gliadin for gluten network, which determine the viscoelastic property of the dough (Sharma *et al.* 2020). Glutenin properties control dough resistance and extensibility (Eagles *et al.* 2006), and gluten elasticity and extensibility have significant influence on dough viscoelasticity (Belderok 2000) and bread-making quality of wheat (Nieto-Talasriz *et al.* 1994). Gluten consists of high-molecular-weight glutenin subunits (HMW-GS) and low-molecular-weight glutenin subunits (LMW-GS) (Lindsay *et al.* 2000). Prolamins are also divided into two classes of monomeric gliadins and polymeric glutenins including both LMW and HMW proteins. These gradually accumulating storage proteins provide necessary nutrients for seed germination and seedling growth, and are the main nutritional components of grains consumed by humans or other animals.

## Conclusions

The current study discovered organ-specific expressions of many important wheat proteins that may contribute to tiller and spike formation as well as kernel development. The data resources of this study may be used to support future analysis of selected genes or proteins and determine how they are linked to grain yield. Future trials should follow up with validations on the putative candidate genes for their precise effect on plant development processes such as tillering, spike formation and kernel development that are important to grain yield. The data will be a valuable resource for future studies on wheat systems biology and integrated, and enhancing agronomically important traits in wheat breeding programmes.

## Supporting Information

The following additional information is available in the online version of this article—

**Table S1.** List of all peptides identified by liquid chromatography-tandem mass spectrometry (LC-MS/MS) analysis with their mapped proteins (both protein group IDs and leading protein ID), molecular weights, sequence start and end position, posterior error probability (PEP), charge state and intensities across samples are provided. Protein group IDs are the identifier(s) of proteins in the group that are identified by both unique and shared peptides and are sorted by the number of identified peptides in a descending order. Leading protein IDs are the proteins that have at least half of the unique plus shared peptides mapped.

**Table S2.** The intensity of all the proteins identified in the seven sampled tissues and associated encoding genes for the identified proteins.

**Table S3.** The proteins along with the genes coding them which were recorded for each sampled tissue. This information corresponds with the Venn diagrams in Fig. 5.

**Table S4.** Differentially abundant proteins (DAPs) for tiller sample and spike initiation site compared to leaf tissue as well as the three kernel samples (5, 10 and 15 days after anthesis (DAA)) compared to ovary tissue.

plaa042_suppl_Supplementary_Table_S1

plaa042_suppl_Supplementary_Table_S2

plaa042_suppl_Supplementary_Table_S3

plaa042_suppl_Supplementary_Table_S4

## Data Availability

The raw MS/MS data were submitted to MASSIVE Data Repository (https://massive.ucsd.edu/), and can be accessible under accession number MSV000083833.

## References

[CIT0001] Acreche M, Slafer GA 2006 Grain weight response to increases in number of grains in wheat in a Mediterranean area. Field Crops Research 98:52–59.

[CIT0002] Aisawi K, Reynolds M, Singh R, Foulkes M 2015 The physiological basis of the genetic progress in yield potential of CIMMYT spring wheat cultivars from 1966 to 2009. Crop Science 55:1749–1764.

[CIT0003] Appels R, Eversole K, Stein N, Feuillet C, Keller B, Rogers J, Pozniak CJ, Choulet F, Distelfeld A, Poland J, Ronen G, Sharpe AG, Barad O, Baruch K, Keeble-Gagnère G, Mascher M, Ben-Zvi G, Josselin A-A, Himmelbach A, Balfourier F, Gutierrez-Gonzalez J, Hayden M, Koh C, Muehlbauer G, Pasam RK, Paux E, Rigault P, Tibbits J, Tiwari V, Spannag M, Lang D, Gundlach H, Haberer G, Mayer KFX, Ormanbekova D, Prade V, Šimková H, Wicker T, Swarbreck D, Rimbert H, Felder M, Guilhot N, Kaithakottil G, Keilwagen J, Leroy P, Lux T, Twardziok S, Venturini L, Juhász A, Abrouk M, Fischer I, Uauy C, Borrill P, Ramirez-Gonzalez RH, Arnaud D, Chalabi S, Chalhoub B, Cory A, Datla R, Davey MW, Jacobs J, Robinson SJ, Steuernage B, van Ex F, Wulff BBH, Benhamed M, Bendahmane A, Concia L, Latrasse D, Bartoš J, Bellec A, Berges H, Doleže J, Frenkel Z, Gill B, Korol A, Letellier T, Olsen O-A, Singh K, Valárik M, Vossen EVD, Vautrin S, Weining S, Fahima T, Glikson V, Raats D, Číhalíková J, Toegelová H, Vrána J, Sourdille P, Darrier B, Barabaschi D, Cattivelli L, Hernandez P, Galvez S, Budak H, Jones JDG, Witek K, Yu G, Small I, Melonek J, Zhou R, Belova T, Kanyuka K, King R, Nilsen K, Walkowiak S, Cuthbert R, Knox R, Wiebe K, Xiang D, Rohde A, Golds T, Čížková J, Akpinar BA, Biyiklioglu S, Gao L, N’Daiye A, Kubaláková M, Šafář J, Alfama F, Adam-Blondon A-F, Flores R, Guerche C, Loaec M, Quesneville H, Condie J, Ens J, Maclachlan R, Tan Y, Alberti A, Aury J-M, Barbe V, Couloux A, Cruaud C, Labadie K, Mangenot S, Wincker P, Kaur G, Luo M, Sehgal S, Chhuneja P, Gupta OP, Jindal S, Kaur P, Malik P, Sharma P, Yadav B, Singh NK, Khurana JP, Chaudhary C, Khurana P, Kumar V, Mahato A, Mathur S, Sevanthi A, Sharma N, Tomar RS, Holušová K, Plíhal O, Clark MD, Heavens D, Kettleborough G, Wright J, Balcárková B, Hu Y, Salina E, Ravin N, Skryabin K, Beletsky A, Kadnikov V, Mardanov A, Nesterov M, Rakitin A, Sergeeva E, Handa H, Kanamori H, Katagiri S, Kobayashi F, Nasuda S, Tanaka T, Wu J, Cattonaro F, Jiumeng M, Kugler K, Pfeifer M, Sandve S, Xun X, Zhan B, Batley J, Bayer PE, Edwards D, Hayashi S, Tulpová Z, Visendi P, Cui L, Du X, Feng K, Nie X, Tong W, Wang L 2018 Shifting the limits in wheat research and breeding using a fully annotated reference genome. Science 4:628. 10.1126/science.aar7191 30115783

[CIT0004] Barrero JM, Mrva K, Talbot MJ, White RG, Taylor J, Gubler F, Mares DJ 2013 Genetic, hormonal, and physiological analysis of late maturity α-amylase in wheat. Plant Physiology 161:1265–1277. 23321420 10.1104/pp.112.209502 PMC3585595

[CIT0005] Barrero-Sicilia C, Hernando-Amado S, González-Melendi P, Carbonero P 2011 Structure, expression profile and subcellular localisation of four different sucrose synthase genes from barley. Planta 234:391–403. 21505865 10.1007/s00425-011-1408-x

[CIT0006] Belderok B 2000 Developments in bread-making processes. 4. Survey of gluten proteins and wheat starches. Plant Foods for Human Nutrition 55:30–39. 10.1023/a:1008199314267 10823487

[CIT0007] Bhave M, Morris CF 2008 Molecular genetics of puroindolines and related genes: allelic diversity in wheat and other grasses. Plant Molecular Biology 66:205–219. 18049798 10.1007/s11103-007-9263-7

[CIT0008] Bondarenko PV, Chelius D, Shaler TA 2002 Identification and relative quantitation of protein mixtures by enzymatic digestion followed by capillary reversed-phase liquid chromatography-tandem mass spectrometry. Analytical Chemistry 74:4741–4749. 12349978 10.1021/ac0256991

[CIT0009] Bottomley RC, Kearns HF, Schofield JD 1982 Characterization of wheat flour and gluten proteins using buffers containing sodium dodecyl sulphate. Journal of the Science of Food and Agriculture 33:4481–4491.

[CIT0010] Bustos-Korts D, Hasan A, Reynolds M, Calderini D 2013 Combining high grain number and weight through a DH-population to improve grain yield potential of wheat in high-yielding environments. Field Crops Research 145:106–115.

[CIT0011] Calderini D, Reynolds M, Slafer GA 2006 Source–sink effects on grain weight of bread wheat, durum wheat, and triticale at different locations. Australian Journal of Agricultural Research 57:227–233.

[CIT0012] Castillo FM, Canales J, Claude A, Calderini DF 2018 Expansin genes expression in growing ovaries and grains of sunflower are tissue-specific and associate with final grain weight. BMC Plant Biology 18:327. 30514222 10.1186/s12870-018-1535-7 PMC6280438

[CIT0013] Chapman JA, Mascher M, Buluç A, Barry K, Georganas E, Session A, Strnadova V, Jenkins J, Sehgal S, Oliker L, Schmutz J, Yelick KA, Scholz U, Waugh R, Poland JA, Muehlbauer GJ, Stein N, Rokhsar DS 2015 A whole-genome shotgun approach for assembling and anchoring the hexaploid bread wheat genome. Genome Biology 16:26. 25637298 10.1186/s13059-015-0582-8 PMC4373400

[CIT0014] Chourey PS, Jain M, Li QB, Carlson SJ 2006 Genetic control of cell wall invertases in developing endosperm of maize. Planta 223:159–167. 16025339 10.1007/s00425-005-0039-5

[CIT0015] Cosgrove DJ 2000 Loosening of plant cell walls by expansins. Nature 407:321–326. 11014181 10.1038/35030000

[CIT0016] Curtis T, Halford NG 2014 Food security: the challenge of increasing wheat yield and the importance of not compromising food safety. The Annals of Applied Biology 164:354–372. 25540461 10.1111/aab.12108 PMC4240735

[CIT0017] Dixon LE, Greenwood JR, Bencivenga S, Zhang P, Cockram J, Mellers G, Ramm K, Cavanagh C, Swain SM, Boden SA 2018 TEOSINTE BRANCHED1 regulates inflorescence architecture and development in bread wheat (*Triticum aestivum*). The Plant Cell 30:563–581. 29444813 10.1105/tpc.17.00961 PMC5894836

[CIT0018] Doebley J, Stec A, Hubbard L 1997 The evolution of apical dominance in maize. Nature 386:485–488. 9087405 10.1038/386485a0

[CIT0019] Dong K, Zhen S, Cheng Z, Cao H, Ge P, Yan Y 2015 Proteomic analysis reveals key proteins and phosphoproteins upon seed germination of wheat (*Triticum aestivum* L.). Frontiers in Plant Science 6:1017. 26635843 10.3389/fpls.2015.01017 PMC4649031

[CIT0020] Duncan O, Trösch J, Fenske R, Taylor NL, Millar AH 2017 Resource: Mapping the *Triticum aestivum* proteome. The Plant Journal: for Cell and Molecular Biology 89:601–616. 27775198 10.1111/tpj.13402

[CIT0021] Dupont FM, Altenbach SB 2003 Molecular and biochemical impacts of environmental factors on wheat grain development and protein synthesis. Journal of Cereal Science 38:133–146.

[CIT0022] Dwivedi S, Sahrawat K, Upadhyaya H, Ortiz R 2013 Food, Nutrition and agrobiodiversity under global climate change. Advances in Agronomy 120:1–128.

[CIT0023] Eagles HA, Cane K, Eastwood RF, Hollamby GJ, Kuchel H, Martin PJ, Cornish GB 2006 Contributions of glutenin and puroindoline genes to grain quality traits in southern Australian wheat breeding programs. Australian Journal of Agricultural Research 57:179–186.

[CIT0024] Edurne BF, Francisco JM, Takayo S, Milagros RL, Takashi A, Javier PR 2003 Sucrose synthase catalyzes the de novo production of ADPglucose linked to starch biosynthesis in heterotrophic tissues of plants. Plant and Cell Physiology 44:500–509. 12773636 10.1093/pcp/pcg062

[CIT0025] Emes MJ, Bowsher CG, Hedley C, Burrell MM, Scrase-Field ES, Tetlow IJ 2003 Starch synthesis and carbon partitioning in developing endosperm. Journal of Experimental Botany 54:569–575. 12508067 10.1093/jxb/erg089

[CIT0026] Fahy B, Siddiqui H, David LC, Powers SJ, Borrill P, Uauy C, Smith AM 2018 Final grain weight is not limited by the activity of key starch-synthesising enzymes during grain filling in wheat. Journal of Experimental Botany 69:5461–5475. 30165455 10.1093/jxb/ery314 PMC6255701

[CIT0027] Feng N, Song G, Guan J, Chen K, Jia M, Huang D, Wu J, Zhang L, Kong X, Geng S, Liu J, Li A, Mao L 2017 Transcriptome profiling of wheat inflorescence development from spikelet initiation to floral patterning identified stage-specific regulatory genes. Plant Physiology 174:1779–1794. 28515146 10.1104/pp.17.00310 PMC5490901

[CIT0028] Ferrante A, Savin R, Slafer G 2012 Differences in yield physiology between modern, well adapted durum wheat cultivars grown under contrasting conditions. Field Crops Research 136:52–64.

[CIT0029] García GA, Hasan AK, Puhl LE, Reynolds MP, Calderini DF, Miralles DJ 2013 Grain yield potential strategies in an elite wheat double-haploid population grown in contrasting environments. Crop Science 53:2577–2587.

[CIT0030] Giroux M, Morris CF 1997 A glycine to serine change in puroindoline b is associated with wheat grain hardness and low level of starch-surface friabilin. Theoretical and Applied Genetics 95:857–864.

[CIT0031] Giroux MJ, Morris CF 1998 Wheat grain hardness results from highly conserved mutations in the friabilin components puroindoline a and b. Proceedings of the National Academy of Sciences of the United States of America 95:6262–6266. 9600953 10.1073/pnas.95.11.6262 PMC27651

[CIT0032] Glenn GM, Younce FL, Pitts MJ 1991 Fundamental physical properties characterizing the hardness of wheat endosperm. Journal of Cereal Science 13:179–194.

[CIT0033] Hawkesford MJ, Araus JL, Park R, Calderini D, Miralles D, Shen T, Zhang J, Parry MAJ 2013 Prospect of doubling global wheat yields. Food and Energy Security 2:34–48.

[CIT0034] Heberle H, Meirelles GV, da Silva FR, Telles GP, Minghim R 2015 InteractiVenn: a web-based tool for the analysis of sets through Venn diagrams. BMC Bioinformatics 16:169. 25994840 10.1186/s12859-015-0611-3 PMC4455604

[CIT0035] Hirose T, Scofield GN, Terao T 2008 An Expression analysis profile for the entire sucrose synthase gene family in rice. Plant Science 174:534–543.

[CIT0036] Hirose T, Takano M, Terao T 2002 Cell wall invertase in developing rice caryopsis: molecular cloning of OsCIN1 and analysis of its expression in relation to its role in grain filling. Plant & Cell Physiology 43:452–459. 11978873 10.1093/pcp/pcf055

[CIT0037] Huang H, Xie S, Xiao Q, Wei B, Zheng L, Wang Y, Cao Y, Zhang X, Long T, Li Y, Hu Y, Yu G, Liu H, Liu Y, Huang Z, Zhang J, Huang Y 2016 Sucrose and ABA regulate starch biosynthesis in maize through a novel transcription factor, ZmEREB156. Scientific Reports 6:27590. 27282997 10.1038/srep27590 PMC4901336

[CIT0038] Huebbner FR, Wall JS 1976 Fractionation and quantitative differences of glutenin from wheat varieties varying in baking quantity. Cereal Chemistry 53:258–263.

[CIT0039] Hussien A, Tavakol E, Horner DS, Muñoz-Amatriaín M, Muehlbauer GJ, Rossini L 2014 Genetics of tillering in rice and barley. The Plant Genome 7:1–20.

[CIT0040] Irshad A, Guo H, Zhang S, Gu J, Zhao L, Xie Y, Xiong H, Zhao S, Ding Y, Ma Y, Liu L 2019 EcoTILLING reveals natural allelic variations in starch synthesis key gene TaSSIV and its haplotypes associated with higher thousand grain weight. Genes (Basel) 10:307. 10.3390/genes10040307 PMC6523294 31003564

[CIT0041] Jiang D, Cao W, Dai T, Jing Q 2003 Activities of key enzymes for starch synthesis in relation to growth of superior and inferior grains on winter wheat (*Triticum aestivum* L.) spike. Plant Growth Regulation 41:247–257.

[CIT0042] Jiang Q, Hou J, Hao C, Wang L, Ge H, Dong Y, Zhang X 2011 The wheat (*T. aestivum*) sucrose synthase 2 gene (TaSus2) active in endosperm development is associated with yield traits. Functional & Integrative Genomics 11:49–61. 20821031 10.1007/s10142-010-0188-x

[CIT0043] Jiao Y, Lee YK, Gladman N, Chopra R, Christensen SA, Regulski M, Burow G, Hayes C, Burke J, Ware D, Xin Z 2018 MSD1 regulates pedicellate spikelet fertility in sorghum through the jasmonic acid pathway. Nature Communications 9:822. 10.1038/s41467-018-03238-4 PMC5826930 29483511

[CIT0044] Jun Z, Weijie H, Zhu P, Hui Z, Feng L, Jialing Y 2019 A putative AGO protein, OsAGO17, positively regulates grain size and grain weight through OsmiR397b in rice. Plant Biotechnology 18. doi:10.1111/pbi.13256. PMC7061870 31529568

[CIT0045] Kapoor KN, Barry DT, Rees RC, Dodi IA, McArdle SE, Creaser CS, Bonner PL 2009 Estimation of peptide concentration by a modified bicinchoninic acid assay. Analytical Biochemistry 393:138–140. 19539599 10.1016/j.ab.2009.06.016

[CIT0046] Komatsuda T, Pourkheirandish M, He C, Azhaguvel P, Kanamori H, Perovic D, Stein N, Graner A, Wicker T, Tagiri A, Lundqvist U, Fujimura T, Matsuoka M, Matsumoto T, Yano M 2007 Six-rowed barley originated from a mutation in a homeodomainleucine zipper I-class homeobox gene. Proceedings of the National Academy of Sciences of the United States of America 104:424–1429. 17220272 10.1073/pnas.0608580104 PMC1783110

[CIT0047] Lewis JM, Mackintosh CA, Shin S, Gilding E, Kravchenko S, Baldridge G, Zeyen R, Muehlbauer GJ 2008 Overexpression of the maize Teosinte Branched1 gene in wheat suppresses tiller development. Plant Cell Reports 27:1217–1225. 18392625 10.1007/s00299-008-0543-8

[CIT0048] Li S 2015 The *Arabidopsis thaliana* TCP transcription factors: a broadening horizon beyond development. Plant Signaling & Behavior 10:e1044192. 26039357 10.1080/15592324.2015.1044192 PMC4622585

[CIT0049] Li X, Qian Q, Fu Z, Wang Y, Xiong G, Zeng D, Wang X, Liu X, Teng S, Hiroshi F, Yuan M, Luo D, Han B, Li J 2003 Control of tillering in rice. Nature 422:618–621. 12687001 10.1038/nature01518

[CIT0050] Lillemo M, Chen F, Xia X, William M, Peña R, Trethowan R, He Z 2006 Puroindoline grain hardness alleles in CIMMYT bread wheat. Journal of Cereal Science 44:86–92.

[CIT0051] Lin Z, Ni Z, Zhang Y, Yao Y, Wu H, Sun Q 2005 Isolation and characterization of 18 genes encoding α- and β-expansins in wheat (*Triticum aestivum* L.). Molecular Genetics and Genomics 274:548–556. 16270219 10.1007/s00438-005-0029-0

[CIT0052] Lindsay MP, Tamas L, Appels R, Skerritt JH 2000 Direct evidence that the number and location of cysteine residues affect glutenin polymer structure. Journal of Cereal Science 31:321–333.

[CIT0053] Liu X, Fu J, Gu D, Liu W, Liu T, Peng Y, Wang J, Wang G 2008 Genome-wide analysis of gene expression profiles during the kernel development of maize (*Zea mays* L.). Genomics 91:378–387. 18280698 10.1016/j.ygeno.2007.12.002

[CIT0054] Lizana XC, Riegel R, Gomez LD, Herrera J, Isla A, McQueen-Mason SJ, Calderini DF 2010 Expansins expression is associated with grain size dynamics in wheat (*Triticum aestivum* L.). Journal of Experimental Botany 61:1147–1157. 20080826 10.1093/jxb/erp380 PMC2826655

[CIT0055] Ma D, Yan J, He Z, Wu L, Xia X 2012 Characterization of a cell wall invertase gene TaCwi-A1 on common wheat chromosome 2A and development of functional markers. Molecular Breeding 29:43–52.

[CIT0056] Ma C, Zhou J, Chen G, Bian Y, Lv D, Li X 2014 iTRAQ-based quantitative proteome and phosphoprotein characterization reveals the central metabolism changes involved in wheat grain development. BMC Genomics 15:1029. doi:10.1186/1471-2164-15-1029. 25427527 PMC4301063

[CIT0057] Marowa P, Ding A, Kong Y 2016 Expansins: roles in plant growth and potential applications in crop improvement. Plant Cell Reports 35:949–965. 26888755 10.1007/s00299-016-1948-4 PMC4833835

[CIT0058] Matus-Cadiz MA, Pozniak CJ, Hucl PJ 2008 Puroindoline allele diversity in Canadian and northern US hard spring wheat varieties differing in kernel hardness. Canadian Journal of Plant Science 88:873–883.

[CIT0060] Merlino M, Leroy P, Chambon C, Branlard G 2009 Mapping and proteomic analysis of albumin and globulin proteins in hexaploid wheat kernels (*Triticum aestivum* L.). Theoretical and Applied Genetics 118:1321–1337. 19277600 10.1007/s00122-009-0983-8

[CIT0061] Miralles DJ, Slafer GA 1995 Yield, biomass and yield components in dwarf, semi-dwarf and tall isogenic lines of spring wheat under recommended and late sowing dates. Plant Breeding 114:392–396.

[CIT0062] Nieto-Taladriz MT, Perretant MR, Rousset M 1994 Effect of gliadins and HMW and LMW subunits of glutenin on dough properties in the F6 recombinant inbred lines from bread wheat cross. Theoretical and Applied Genetics 88:81–88. 24185886 10.1007/BF00222398

[CIT0063] Parry MA, Reynolds M, Salvucci ME, Raines C, Andralojc PJ, Zhu XG, Price GD, Condon AG, Furbank RT 2011 Raising yield potential of wheat. II. Increasing photosynthetic capacity and efficiency. Journal of Experimental Botany 62:453–467. 21030385 10.1093/jxb/erq304

[CIT0064] Pask A, Reynolds M 2013 Breeding for yield potential has increased deep soil water extraction capacity in irrigated wheat. Crop Science 53:2090–2104.

[CIT0065] Polpitiya AD, Qian WJ, Jaitly N, Petyuk VA, Adkins JN, Camp DG 2nd, Anderson GA, Smith RD 2008 DAnTE: a statistical tool for quantitative analysis of -omics data. Bioinformatics 24:1556–1558. 18453552 10.1093/bioinformatics/btn217 PMC2692489

[CIT0066] Ramsay L, Comadran J, Druka A, Marshall DF, Thomas WT, Macaulay M, MacKenzie K, Simpson C, Fuller J, Bonar N, Hayes PM, Lundqvist U, Franckowiak JD, Close TJ, Muehlbauer GJ, Waugh R 2011 INTERMEDIUM-C, a modifier of lateral spikelet fertility in barley, is an ortholog of the maize domestication gene TEOSINTE BRANCHED 1. Nature Genetics 43:169–172. 21217754 10.1038/ng.745

[CIT0067] Reynolds M, Foulkes J, Furbank R, Griffiths S, King J, Murchie E, Parry M, Slafer G 2012 Achieving yield gains in wheat. Plant, Cell & Environment 35:1799–1823. 10.1111/j.1365-3040.2012.02588.x 22860982

[CIT0068] Sadras V, Lawson C 2011 Genetic gain in yield and associated changes in phenotype, trait plasticity and competitive ability of South Australian wheat varieties released between 1958 and 2007. Crop and Pasture Science 62:533–549.

[CIT0069] Schilling S, Pan S, Kennedy A, Melzer R 2018 MADS-box genes and crop domestication: the jack of all traits. Journal of Experimental Botany 69:1447–1469. 29474735 10.1093/jxb/erx479

[CIT0070] Serrago AR, Alzueta I, Savin R, Slafer GA 2013 Understanding grain yield responses to source–sink ratios during grain filling in wheat and barley under contrasting environments. Field Crops Research 150:42–51.

[CIT0071] Sharma A, Garg S, Sheikh I, Vyas P, Dhaliwal HS 2020 Effect of wheat grain protein composition on end-use quality. Journal of Food Science and Technology 57:2771–2785. doi:10.1007/s13197-019-04222-6. 32624587 PMC7316921

[CIT0072] Shiferaw B, Amale M, Braun HJ, Duveiller E, Reynolds M, Muricho G 2010 Crops the feed the world 10. Past successes and future challenges to the role played by wheat in global food security. Food Security 5:291–317.

[CIT0073] Silva MP, Ricardo CPP 1992 β-Fructosidases and in vitro dedifferentiation-redifferentiation of carrot cells. Phytochemistry 31:1507–1511.

[CIT0074] Singh M, Singh S, Randhawa H, Singh J 2013 Polymorphic homoeolog of key gene of RdDM pathway, ARGONAUTE4_9 class is associated with pre-harvest sprouting in wheat (*Triticum aestivum* L.). PLoS ONE 8:e77009. 24130825 10.1371/journal.pone.0077009 PMC3793957

[CIT0075] Spielbauer G, Margl L, Hannah LC, Römisch W, Ettenhuber C, Bacher A, Gierl A, Eisenreich W, Genschel U 2006 Robustness of central carbohydrate metabolism in developing maize kernels. Phytochemistry 67:1460–1475. 16815503 10.1016/j.phytochem.2006.05.035

[CIT0076] Sreenivasulu N, Schnurbusch T 2012 A genetic playground for enhancing grain number in cereals. Trends in Plant Science 17:91–101. 22197176 10.1016/j.tplants.2011.11.003

[CIT0077] Sturm A 1999 Invertases. Primary structures, functions, and roles in plant development and sucrose partitioning. Plant Physiology 121:1–8. 10482654 10.1104/pp.121.1.1 PMC1539224

[CIT0078] Subbaiah CC, Huber SC, Sachs MM, Rhoads D 2007 Sucrose synthase: expanding protein function. Plant Signaling & Behavior 2:28–29. 19704804 10.4161/psb.2.1.3646 PMC2633894

[CIT0079] Thévenot C, Laurière C, Mayer C, Simond-Côte E, Daussant J 1992 α-Amylase changes during development and germination of maize kernels. Journal of Plant Physiology 140:61–65.

[CIT0080] Wang L, Ruan YL 2012 New insights into roles of cell wall invertase in early seed development revealed by comprehensive spatial and temporal expression patterns of GhCWIN1 in cotton. Plant Physiology 160:777–787. 22864582 10.1104/pp.112.203893 PMC3461555

[CIT0081] Wang YQ, Wei XL, Xu HL, Chai CL, Meng K, Zhai HL, Sun AJ, Peng YG, Wu B, Xiao GF, Zhu Z 2008 Cell-wall invertases from rice are differentially expressed in caryopsis during the grain filling stage. Journal of Integrative Plant Biology 50:466–474. 18713381 10.1111/j.1744-7909.2008.00641.x

[CIT0082] Weschke W, Panitz R, Gubatz S, Wang Q, Radchuk R, Weber H, Wobus U 2003 The role of invertases and hexose transporters in controlling sugar ratios in maternal and filial tissues of barley caryopses during early development. The Plant Journal 33:395–411. 12535352 10.1046/j.1365-313x.2003.01633.x

[CIT0083] Xu T, Bian N, Wen M, Xiao J, Yuan C, Cao A, Zhang S, Wang X 2017 Characterization of a common wheat (*Triticum aestivum* L.) high-tillering dwarf mutant. Theoretical and Applied Genetics 130:483–494. 27866225 10.1007/s00122-016-2828-6

[CIT0084] Yadhu S, Anil KG, Achla S, Navtej SB 2015 Differential response of wild and cultivated wheats to water deficits during grain development: changes in soluble carbohydrates and invertases. Physiology and Molecular Biology of Plants 21:169–177. 25964711 10.1007/s12298-015-0283-5 PMC4411390

[CIT0085] Yang M, Dong J, Zhao W, Gao X 2016a Characterization of proteins involved in early stage of wheat grain development by iTRAQ. Journal of Proteomics 136:157–166. 26779988 10.1016/j.jprot.2016.01.002

[CIT0086] Yang M, Gao X, Dong J, Gandhi N, Cai H, Von Wettstein DH, Rustgi S, Wen S 2017 Pattern of protein expression in developing wheat grains identified through proteomic analysis. Frontiers of Plant Science 8:962. 10.3389/fpls.2017.00962 PMC5465268 28649254

[CIT0087] Yang Y, Yu Y, Bi W, Kang Z 2016b Quantitative proteomics reveals the defense response of wheat against *Puccinia striiformis* f. sp. *tritici*. Scientific Reports 6:34261. 27678307 10.1038/srep34261 PMC5039691

[CIT0088] Zhang N, Chen F, Huo W, Cui D 2015 Proteomic analysis of middle and late stages of bread wheat (*Triticum aestivum* L.) grain development. Frontiers in Plant Science 6:735. 26442048 10.3389/fpls.2015.00735 PMC4569854

